# Editorial: New advancement in tumor microenvironment remodeling and cancer therapy, volume II

**DOI:** 10.3389/fcell.2026.1859833

**Published:** 2026-05-06

**Authors:** Sumei Qin, Xiaoxiong Xiao, Ying Shen, Zhihua Pei, Xiangsheng Zuo, Yi Yao

**Affiliations:** 1 Cancer Center, Renmin Hospital of Wuhan University, Wuhan, China; 2 Hubei Provincial Research Center for Precision Medicine of Cancer, Wuhan, China; 3 Department of Thoracic Surgery, Xiangya Hospital, Central South University, Changsha, China; 4 Sun Yat-sen University Cancer Center, Guangzhou, China; 5 Hubei Key laboratory of Agriculture bioinformatics, Collage of informatics, Huazhong Agriculture University, Wuhan, China; 6 Department of Gastrointestinal Medical Oncology, University of Texas MD Anderson Cancer Center, Houston, TX, United States

**Keywords:** tumor microenvironment (TME), cancer-associated fibroblasts (CAFs), immune checkpoint therapy (ICT), precision oncology, spatial transcriptomics

The tumor microenvironment (TME) is a complex ecosystem composed of diverse cellular components (including fibroblasts, immune cells, and endothelial cells) and non-cellular elements (such as extracellular matrix and cytokines). Although traditionally considered a passive bystander in tumorigenesis, the TME is now recognized as a central driver of tumor progression, metastasis, immune evasion, and therapeutic resistance. This paradigm shift—from “bystander” to “participant” and further to “regulator”—marks a new era in tumor biology (Grant and Ferrer, 2025). Understanding TME remodeling mechanisms not only provides novel insights into malignant tumor behavior but also opens broad horizons for developing innovative therapeutic strategies that move beyond the traditional tumor-centric view.

Recent studies have dissected the heterogeneity of cancer-associated fibroblasts (CAFs) at an unprecedented resolution, identifying functionally distinct subsets such as myofibroblastic CAFs (myCAFs) and inflammatory CAFs (iCAFs). These subsets play different, and even opposing, roles in tumor progression and can serve as biomarkers for predicting prognosis and immunotherapy response (Cords et al., 2024). For instance, Chen et al. identified a PRRX2+ myCAF subset that promotes perineural invasion via TGF-β signaling in colorectal cancer, closely correlating with poor prognosis. Similarly, Damisch et al. revealed fibromuscular cell heterogeneity in prostate cancer stroma with clinical correlates. Notably, driven by advances in single-cell sequencing and spatial omics technologies, an increasing number of novel CAF subsets have been identified. For example, a chemotherapy-induced PTGER3^+^ lipoCAF subset has been shown to produce the lipid metabolite 11-HETE, thereby enhancing CD8^+^ T cell function (Ma et al., 2026).

The mechanisms underlying dynamic immune microenvironment remodeling are becoming increasingly clear. Tumor cells establish a potent immunosuppressive network through chemokine secretion, immune checkpoint molecule expression, and metabolic reprogramming, recruiting regulatory T cells (Tregs) and myeloid-derived suppressor cells (MDSCs), and inducing M2 polarization of tumor-associated macrophages (TAMs) (Zhang et al., 2025). In addition to tumor cell-intrinsic drivers, chemotherapeutic intervention is capable of profoundly reshaping the immune microenvironment. Gemcitabine, for instance, is not merely a conventional cytotoxic chemotherapeutic agent, but also exhibits immunomodulatory properties that may potentiate anti-tumor immune responses when combined with immune checkpoint inhibitors (Principe et al., 2020; Ho et al., 2020). However, subsequent studies have shown that this immunomodulation is not unidirectional. As reviewed by Nemati et al., gemcitabine exhibits dual immunomodulatory roles, activating immunity via immunogenic cell death while potentially promoting M2 polarization or MDSC accumulation, highlighting the complex interplay between therapy and TME. Collectively, these effects of gemcitabine reveal the plasticity of TME and highlight the potential of immunotherapy strategies in regulating the TME.

Beyond cellular components, metabolic and physical factors within the TME are also research hotspots. Hypoxia, a hallmark of most solid tumors, drives hypoxia-inducible factor (HIF) signaling that promotes angiogenesis and reprograms metabolism, exacerbating immunosuppression. Wang et al. found that overexpression of VWF, a malignant gene in tumor-associated endothelial cells, drives hypoxic metabolism in gastric cancer and fosters an immunosuppressive microenvironment, thereby diminishing the efficacy of immunotherapy. Hou et al. demonstrated that insufficient radiofrequency ablation (IRFA) for hepatocellular carcinoma promotes malignant progression of residual tumors through local inflammation, hypoxia, non-coding RNA dysregulation, and autophagy. Additionally, Chen et al. uncovered disulfidptosis, a novel cell death triggered by glucose starvation in SLC7A11-high ovarian cancer cells, offering new strategies for targeting metabolic vulnerabilities in the TME.

Emerging frontiers further enrich our understanding of TME complexity. Spatial transcriptomics has revealed that TME composition varies drastically between tumor core and invasive front, with distinct CAF and immune cell distributions that influence therapeutic response (Ji et al., 2025). Spatial multi-omics analyses of the cellular neighborhood of CAFs have further identified distinct spatial CAF subtypes characterized by unique organization, expression profiles, and interactions. These subtypes are conserved across cancer types and associate with distinct TME features and clinical outcomes, underscoring that CAF phenotypes and functions are shaped by neighboring cell interactions (Liu et al., 2025). Additionally, the senescence-associated secretory phenotype (SASP) derived from senescent stromal cells can paradoxically promote tumor progression and immune-suppression (Cao et al., 2025). Recent studies also highlight that intratumoral microbiota modulate local immune landscapes and impact the response to immune checkpoint inhibitors, further complicating therapeutic strategies (Yan et al., 2026). Furthermore, the establishment of next-generation high-throughput technology platforms has provided novel tools for the spatial dissection of the TME, revealing how the loss of various tumor suppressor genes shapes the TME and contributes to immunotherapy resistance (Wang et al., 2026). Collectively, these advances underscore the need for multi-dimensional approaches to dissect TME complexity (Larson et al., 2025).

Despite these advances, several challenges remain. The foremost challenge is the extreme spatial and temporal heterogeneity of the TME, which makes it difficult for studies based on limited markers to capture the full picture. Our understanding of the dynamic processes governing TME remodeling during tumorigenesis, progression, and treatment remains incomplete. Furthermore, functional redundancy among TME components often renders single-target interventions ineffective, leading to therapeutic resistance (Hanahan et al., 2025). More importantly, immune, stromal, metabolic, and microbial components form a highly coupled regulatory network, while inter-patient heterogeneity further increases the difficulty of precise intervention. Together, these issues continue to constrain the mechanistic dissection of the TME and its translation into clinical practice.

Looking ahead, harnessing TME remodeling for clinical benefit requires more precise, combinatorial, and dynamic strategies. First, advancing precision targeting from the “population” level to the “subset” level is crucial. Developing drugs that specifically eliminate key CAF subsets (e.g., PRRX2+ myCAFs), immunosuppressive cell subsets, or metabolically vulnerable subsets (e.g., SLC7A11-high cells) is a priority. Second, “multi-pronged” combination strategies are essential. Future approaches should rationally combine TME-targeted agents with immunotherapies, chemotherapy, targeted therapy, or local ablation. For example, combining IRFA with anti-inflammatory or immunomodulatory agents could block accelerated progression, as reported by Hou et al. Third, dynamic monitoring and precision stratification using liquid biopsies—such as analyzing fragmentomic features of circulating tumor DNA as shown by Zhang et al.—and artificial intelligence-based multi-modal data integration represent essential paths toward personalized medicine (Luo et al., 2025). Fourth, spatiotemporal analysis represents the next frontier in TME research. The integration of spatial transcriptomics, spatial proteomics, real-time cellular imaging, clinical imaging technologies, and artificial intelligence will deepen our understanding of tissue architecture, cellular interactions, and disease progression, thereby advancing precision medicine toward a more refined characterization of the dynamic and contextual nature of cancer biology (Larson et al., 2025).

In conclusion, research on tumor microenvironment remodeling is transitioning from descriptive phenomenology to mechanistic elucidation and is gradually moving towards clinical application. The future challenge lies in transforming our profound understanding of TME complexity into effective therapeutic strategies capable of precisely and dynamically intervening in the malignant progression of tumors, ultimately yielding tangible survival benefits for patients. The realization of this goal will signify a true paradigm shift in oncology, moving from a “tumor-centric” approach into a new era of “microenvironment-targeted” precision medicine.
