# Correction: Editorial: New advancement in tumor microenvironment remodeling and cancer therapy, volume II

**DOI:** 10.3389/fcell.2026.1878088

**Published:** 2026-05-18

**Authors:** Sumei Qin, Xiaoxiong Xiao, Ying Shen, Zhihua Pei, Xiangsheng Zuo, Yi Yao

**Affiliations:** 1 Cancer Center, Renmin Hospital of Wuhan University, Wuhan, China; 2 Hubei Provincial Research Center for Precision Medicine of Cancer, Wuhan, China; 3 Department of Thoracic Surgery, Xiangya Hospital, Central South University, Changsha, China; 4 Sun Yat-sen University Cancer Center, Guangzhou, China; 5 Hubei Key laboratory of Agriculture bioinformatics, Collage of informatics, Huazhong Agriculture University, Wuhan, China; 6 Department of Gastrointestinal Medical Oncology, University of Texas MD Anderson Cancer Center, Houston, TX, United States

**Keywords:** tumor microenvironment (TME), cancer-associated fibroblasts (CAFs), immune checkpoint therapy (ICT), precision oncology, spatial transcriptomics

An incorrect Funding statement was provided. The correct funding statement reads:

The author(s) declared that financial support was received for this work and/or its publication. YY was supported by the International Scientific Exchange Foundation of China (No. SWYY2025003036) and Beijing Bethune Charitable Foundation (No. 2023-YJ-152-J-001); YS was supported by the National Natural Science Foundation of China (NSFC) grant (82273314).

The original article has been updated.

